# Precision Glyco-Modulation of Macrophages with EF-M2 (Immutalon^TM^) Improves Function and Lowers Inflammatory Biomarkers in Aging Dogs: A Double-Blind, Placebo-Controlled Trial

**DOI:** 10.3390/vetsci12121168

**Published:** 2025-12-09

**Authors:** Evgeny Pokushalov, Dmitry Kudlay, Claire Garcia, John Smith, Nikolai Revkov, Anastasya Shcherbakova, Richard Miller

**Affiliations:** 1 Scientific Research Laboratory, Triangel Scientific, San Francisco, CA 94101, USA; info@triangelcompany.com (C.G.); nikitenko.marina.maratovna@gmail.com (J.S.); arrhythmdoc@gmail.com (R.M.); 2 Center for New Medical Technologies, 630090 Novosibirsk, Russia; 3 Institute of Pharmacy, I.M. Sechenov First Moscow State Medical University (Sechenov University), 119435 Moscow, Russia; d624254@gmail.com; 4 Institute of Medicine and Medical Technologies, Novosibirsk State University, 630090 Novosibirsk, Russia; 5 VEGA Veterinary Clinic, 630049 Novosibirsk, Russia; revkov111@gmail.com; 6 BALTO Veterinary Clinic, 630055 Novosibirsk, Russia; sibirnas@yandex.ru

**Keywords:** CLEC10A, EF-M2 (GcMAF derivative), low-grade inflammation, macrophage modulation, geriatric dogs, inflammaging

## Abstract

We evaluated whether macrophage-targeted glyco-modulation can improve day-to-day function in aging companion animals. In a multicenter, randomized, double-blind, placebo-controlled trial, 60 client-owned geriatric dogs (≥10 years) received subcutaneous EF-M2 (0.1 μg/kg, every 72 h for 4 weeks; protocolized, blinded step-up to every 48 h at day 14 for partial responders) or matched placebo, followed by 4 weeks off-treatment. Two prespecified co-primary endpoints were tested hierarchically: change in accelerometer-measured active minutes/day at week 1 and change in a day-28 vitality composite (z-score of inverted CBPI-PSS, HRQL-vitality, and appetite VAS). EF-M2 was superior to placebo on both: +23.05 min/day at week 1 (95% CI, 18.16–27.94; *p* < 0.001) and +2.01 z-units at day 28 (95% CI, 1.52–2.50; *p* < 0.001). Key secondaries favored EF-M2, including greater week-4 activity (+33.00 min/day, 26.83–39.18; *p* < 0.001), lower BAER auditory threshold (−5.28 dB, −7.53 to −3.04; *p* < 0.001), and reduced transepidermal water loss (−1.35 g/m^2^·h, −2.45 to −0.25; *p* = 0.02); multiplicity was controlled with Holm–Bonferroni procedures. Owner-reported global improvement was more frequent with EF-M2 at day 28 (93.3% vs. 10.0%) and remained higher at day 56 off-treatment (50.0% vs. 16.7%). Prespecified pharmacodynamic markers shifted in directions consistent with M2-skewing (ARG1:iNOS ratio and IL-10 increased; TNF-α decreased) by day 7. Adverse events were infrequent, mild, and similar to placebo; no treatment-related withdrawals or serious events occurred. These findings support macrophage-targeted glyco-modulation as a promising approach to rapidly improve real-world activity and multidomain vitality in older dogs, with short-term signals persisting off-treatment; longer, adequately powered trials are warranted to define durability, structural outcomes, and phenotype-specific benefits.

## Simple Summary

Older dogs often slow down and lose “vitality” because of low-grade, chronic inflammation. EF-M2 is a protein-based treatment designed to gently nudge immune cells called macrophages toward a calmer, repair-oriented state. In a randomized, double-blind, placebo-controlled study of 60 geriatric pet dogs (≥10 years), EF-M2 increased free-living activity and improved owner-reported vitality within a few weeks. On average, dogs receiving EF-M2 added about 23 more active minutes per day during the first treatment week compared with placebo, and they showed broader gains in a prespecified vitality score by day 28; side effects were uncommon and similar to placebo. Some benefits persisted for four weeks after the last dose. These results suggest that precisely targeting innate immunity may help older dogs stay more active and feel better, but longer studies are needed to confirm how durable the effects are and which dogs benefit most.

## 1. Introduction

Late-life functional decline is tightly linked to a chronic, low-grade inflammatory state (“inflammaging”) rooted in myeloid dysregulation [1,2,3]. Innate immune cells—especially tissue macrophages—integrate cytokine and metabolic cues and can perpetuate pain, fatigue, and reduced activity when locked in pro-inflammatory programs [4,5]. Therapies that durably and selectively repolarize macrophages toward reparative states, without broad immunosuppression, are therefore an attractive strategy to restore vitality and physical function in older organisms [6,7]. Precision glyco-engineering of vitamin D-binding protein has recently produced analytically defined ligands (GcMAF2.0 variants) that display a single terminal α-N-acetyl-galactosamine (α-GalNAc) epitope to the C-type lectin receptor CLEC10A (CD301) [8,9,10,11], enabling “dial-a-bias” control over macrophage state with batch-traceable chemistry and receptor potency [12,13,14]. EF-M2 (Immutalon^TM^) represents an M2-skewing lot in this platform, designed to favor IL-10/ARG1 programs while restraining TNF-α/iNOS outputs [15,16].

Early cross-species data support the biological plausibility and safety of this approach. In a rigorously masked, randomized, placebo-controlled trial in client-owned dogs with spontaneous hip or elbow osteoarthritis, subcutaneous EF-M2 produced clinically meaningful improvements in pain and objective locomotor function over four weeks, accompanied by coherent pharmacodynamic shifts toward an M2-dominant serum signature (ARG1/iNOS ↑, IL-10 ↑, TNF-α ↓) and a tolerability profile indistinguishable from placebo—first-in-species evidence that targeted macrophage modulation translates into functional benefit in vivo [15,16]. These results, together with a receptor-to-phenotype mechanistic continuum anchored in CLEC10A biology [8,9,10,11], motivate testing whether systemic macrophage repolarization can also enhance day-to-day “vitality” and activity in aging beyond a single disease model [1,2,3].

The present trial, GERI-VITAL-DOG, prospectively evaluates EF-M2 (Immutalon^TM^) in geriatric companion dogs under a randomized, double-blind, placebo-controlled design with objective actigraphy and owner-reported health status as clinically meaningful outcomes [17,18,19,20]. The protocol prespecifies two co-primary endpoints: (1) change in daily active minutes at day 7 relative to a baseline week and (2) change at day 28 in a standardized “vitality” composite (z-score across CBPI–Pain Severity, HRQL-vitality, and appetite VAS) [17,18,19]. To link clinical effects to mechanism, the protocol integrates a pharmacodynamic panel (ARG1/iNOS ratio, IL-10, TNF-α, and inflammation markers) at multiple time points [16]. Dosing employs short-cycle subcutaneous administration (q72 h for 14 days) with a concealed, protocolized step-up to q48 h in partial responders, while preserving blinding through mirrored procedures in the placebo arm.

Against this background, we hypothesized that short-cycle EF-M2 would increase objective physical activity within one week and improve owner-perceived vitality by four weeks versus placebo, and that these gains would track with a serum shift toward an M2-dominant macrophage signature consistent with EF-M2’s CLEC10A-anchored mechanism. This study was designed to provide randomized, blinded clinical evidence on efficacy, durability off-drug, and pharmacodynamic coherence for a precision macrophage-reprogramming strategy, extending prior osteoarthritis-centered observations to the broader problem of late-life vitality.

Rationale and Significance. By combining an analytically defined macrophage modulator with multimodal endpoints and prespecified mechanistic readouts, this trial addresses a central gap in geroscience therapeutics: whether selective, receptor-guided repolarization of the innate immune compartment can translate into rapid, measurable improvements in everyday function in the aging organism [1,2,3,6,7,8,9,10,11]. The data reported here are intended to inform future structure-validated, longer-duration studies and to clarify exposure–response features (including a step-up paradigm) that would be essential for clinical optimization.

## 2. Materials and Methods

### 2.1. Study Design and Oversight

GERI-VITAL-DOG was a multicenter, randomized, double-blind, placebo-controlled, parallel-group clinical trial in geriatric dogs (1:1 allocation). The finalized protocol (version v1.0; 9 November 2024) prespecified two co-primary endpoints, the timing of assessments, accelerometry quality criteria, and a protocolized dose-escalation (“step-up”) rule at day 14 to preserve equipoise and assay sensitivity. Reporting adhered to CONSORT-Vet/ARRIVE guidance. All analyses followed the protocol and the Statistical Analysis Plan (SAP).

The trial was conducted at two referral veterinary clinics for companion animals. All study operations (design finalization, conduct, monitoring, data management, and statistical analysis) were performed independently by investigators from the Center for New Medical Technologies (Novosibirsk, Russia) and the Triangel Scientific Research Laboratory (San Francisco, CA, USA). The investigational product and unrestricted funding were provided by Activator MAF, LLC (Novosibirsk, Russia), which had no role in the study design, conduct, data collection, analysis, interpretation, or manuscript preparation.

### 2.2. Participants

**Figure 1:**
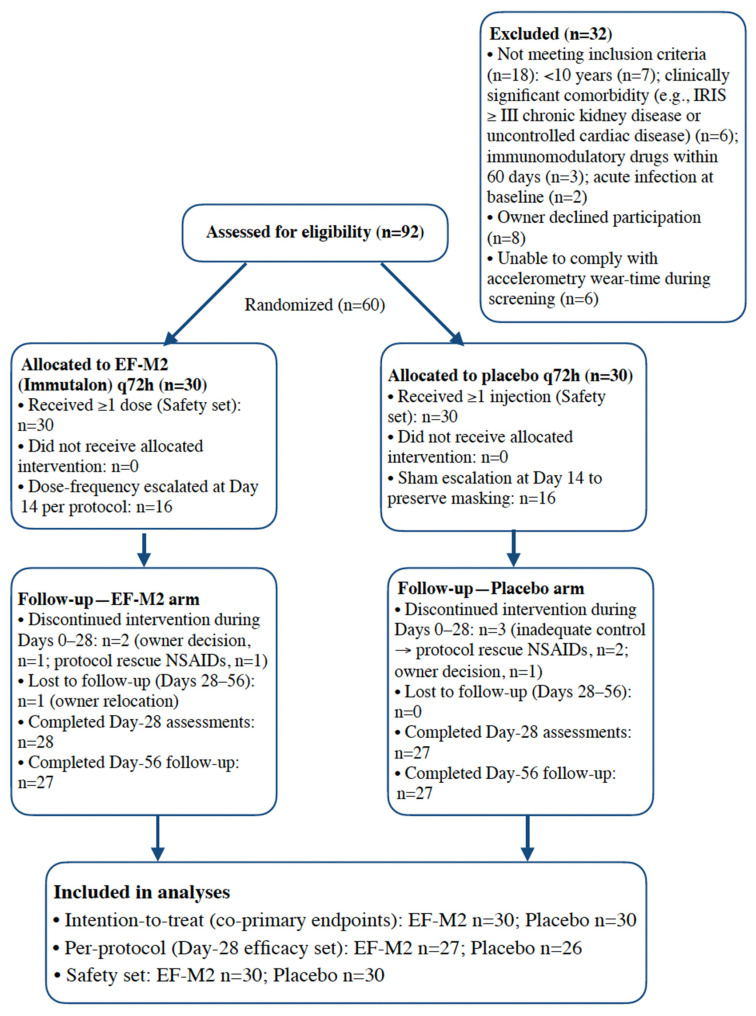
CONSORT-Vet participant flow for GERI-VITAL-DOG.

Client-owned dogs aged ≥10 years were eligible if clinically stable and if owners consented to have the dog wear an accelerometer for ≥20 h/day. Key exclusions included terminal/decompensated disease, recent systemic immunosuppression (<60 days), acute infection, pregnancy/lactation, and conditions precluding valid endpoint assessment. Owners provided written informed consent before any study procedures. Full inclusion/exclusion and analytic populations (intention-to-treat (ITT), per-protocol (PP), safety) were prespecified (Figure 1).

### 2.3. Randomization and Masking

Participants were randomized 1:1 to EF-M2 or placebo using a computer-generated sequence prepared by an independent statistician, with concealed permuted blocks of variable size and stratification by center, age (10–12 vs. >12 years), baseline pain status (CBPI-PSS ≥4: yes/no), and neuter status. Allocation codes were embedded in a secure electronic data-capture system and revealed to the unblinded pharmacist only after eligibility confirmation; investigators, treating veterinarians, owners, and outcome assessors remained unaware of group assignments throughout the trial. To maintain masking, the placebo arm received “mirror” injections (including “mirror” step-up procedures) on the same schedule as the active arm.

Owners were not informed of treatment allocation, did not have access to vial labels or preparation logs, and observed identical injection procedures and schedules in both groups. Any local reactions or transient post-injection behaviors were recorded but were infrequent and balanced across groups, reducing the likelihood of systematic unblinding.

### 2.4. Interventions

Investigational product and vehicle. EF-M2 (Immutalon^TM^, Activator MAF LLC, Novosibirsk, Russia) was supplied as a sterile, preservative-free aqueous solution of GcMAF-derived EF-M2 protein (1 µg/mL) in 0.9% sodium chloride (normal saline) in 1 mL Type I glass vials. For study use, dogs received 0.1 µg/kg (0.1 mL/kg) by subcutaneous injection. The placebo consisted of the identical vehicle (0.9% sodium chloride) without EF-M2, filled into visually indistinguishable vials and administered at the same volume per kilogram and on a schedule mirrored to that of the active regimen, including the protocolized step-up.

Dosing during days 0–14 was every 72 h (q72 h); at day 14, dogs with <25% improvement in their prespecified profile domain (pain when pain predominated; pruritus when pruritus predominated; otherwise the vitality composite) were escalated per protocol to every 48 h (q48 h) through day 28 as part of the randomized treatment strategy. Concomitant care followed site standards, with prespecified restrictions on initiating systemic immunosuppressants.

The 0.1 µg/kg dose and short-cycle subcutaneous schedule were selected based on prior EF-M2 studies in dogs with spontaneous osteoarthritis, in which this dose range and frequency produced reproducible changes in macrophage-related pharmacodynamic markers and clinically meaningful improvements in pain and function with good tolerability [15]. The present regimen (q72 h with protocolized step-up to q48 h in partial responders) was designed to balance sustained CLEC10A engagement with practicality for outpatient administration.

### 2.5. Outcomes

Co-primary endpoints (gatekeeping):

P1 (day 7): Change in objectively measured active minutes/day from the baseline week (days 6…−1) to the treatment window days 1–7.

P2 (day 28): Change in vitality composite z-score from day 0 to day 28. The composite equals the sum of domain-wise z-scores standardized to the day 0 distribution of the full cohort: CBPI-PSS (sign-inverted) [17], HRQL-vitality, and appetite VAS [18,19].

For interpretability, each component score was standardized to the day 0 distribution of the full cohort (mean 0, SD 1) before summation. Thus, a 1-unit increase in the composite corresponds to an improvement of approximately one standard deviation across the three-domain profile, i.e., a dog moving from the cohort average at baseline into a clearly improved range in pain, vitality, and/or appetite.

Key secondary endpoints:

BAER auditory threshold (Δ day 28–day 0; lower is better), TEWL (Δ day 28–day 0; lower is better), objective activity at week 4 (Δ [days 22–28] − baseline), and symptom scales (PVAS for dogs symptomatic with pruritus at baseline; OTIS-3 for those symptomatic with otitis at baseline) [19]. Exploratory endpoints included cognition/vision measures and PGIC at days 28 and 56; mechanistic PD endpoints (ARG1:iNOS ratio, IL-10, TNF-α, hs-CRP, IL-6) were collected at days 0/7/14/28/56. All definitions, windows, and sign conventions were protocol-specified.

### 2.6. Assessments and Quality Control

Accelerometry: Dogs wore a continuously recording device during the baseline week (days 6…−1) and treatment windows (days 1–7 and 22–28) [20]. A valid day required ≥20 h of wear-time and ≤3 non-wear bouts. For an analysis window to be evaluable, ≥5 valid days were required; only valid days contributed to the window means.

Clinical scales: CBPI-PSS, HRQL-vitality, appetite VAS, PVAS, and OTIS-3 were obtained at prespecified visits (days 0, 7, 14, 28, 56) [17,18,19]. Dogs entered PVAS/OTIS-3 analyses only if symptomatic at baseline.

Objective modules: BAER thresholds (dB) and TEWL (g/m^2^·h; standardized skin sites) were measured per protocol at baseline and day 28 (and optionally day 56 for durability).

Pharmacodynamic laboratory methods: Venous blood for pharmacodynamic analyses (ARG1, iNOS, IL-10, TNF-α, hs-CRP, IL-6) was collected at days 0, 7, 14, 28, and 56 into serum separator tubes, centrifuged within 60 min, and serum aliquots were stored at −80 °C until batch analysis at a central laboratory. All pharmacodynamic assays were performed by personnel blinded to treatment allocation. Serum IL-10, TNF-α, and IL-6 were quantified using commercially available canine-specific sandwich ELISA kits run in duplicate according to the manufacturer’s instructions; hs-CRP was measured with a high-sensitivity canine CRP ELISA; and ARG1 and iNOS were quantified using species-validated ELISA kits, from which the prespecified ARG1:iNOS ratio was calculated for each dog and time point. Standard curves were fitted using four-parameter logistic regression, and internal quality-control samples were included on each plate to ensure intra- and inter-assay variability <15%.

Safety: Adverse events (AEs) and serious AEs (SAEs) were captured at each visit and graded per VCOG-CTCAE; temperature and local injection-site reactions were systematically recorded. Laboratory monitoring (ALT/AST, renal indices) occurred at a minimum at day 28.

### 2.7. Sample-Size Calculation

The target enrollment was n = 60 (30/group). Based on the co-primary P1 endpoint, a between-group difference of ~12–15 min/day in active minutes with SD ≈ 20 was expected to yield ≥ 80% power at α = 0.05 with ~27 dogs per group; this was rounded to 30 per group. A blinded variance re-estimation after ≥50% enrollment was permitted without α inflation.

### 2.8. Statistical Analysis

Analyses used the ITT population for primary inference; PP analyses were prespecified as sensitivity. All tests were 2-sided with α = 0.05; effect estimates are reported with 95% CIs and exact *p*-values.

- Co-primary endpoints: For P1 and P2, an ANCOVA model of change from baseline was fitted with treatment group as a fixed effect and the corresponding baseline value as a covariate; additional fixed effects for center/strata could be included as prespecified sensitivity terms. The hierarchical gatekeeping sequence required statistical significance on P1 before formal testing of P2.
- Key secondary endpoints: Continuous endpoints (e.g., week-4 activity, BAER, TEWL) were analyzed via ANCOVA on change from baseline with baseline as covariate. Family-wise error was controlled across key secondaries using Holm–Bonferroni procedures; BAER was a prioritized secondary endpoint. Symptom endpoints (PVAS, OTIS-3) were restricted to dogs symptomatic at baseline per protocol.
- Responder endpoints: PGIC responder analyses used prespecified cut points; between-group comparisons used Fisher’s exact test with 95% CIs for risk differences.
- Pharmacodynamic endpoints: Δ(day 7–day 0) changes were compared between groups using ANCOVA, adjusting for baseline; exploratory correlations of ΔPD with clinical changes and mediation analyses were prespecified in the SAP.
- Missing data: Primary analyses assumed missing at random (MAR) and used ANCOVA/MMRM as appropriate; sensitivity analyses used multiple imputation (MICE) and PP populations.

Treatment-policy principle: The day 14 step-up algorithm was part of the randomized treatment strategy; all post-step-up data were analyzed under ITT without adjustment for post-randomization treatment modifications.

### 2.9. Data Monitoring and Quality Assurance

An electronic data capture (EDC) system enforced range/timing checks, accelerometry wear-time validations, and windowing rules; centralized monitoring and on-site visits were conducted per protocol and standard operating procedures. Specimens were archived at −80 °C and shipped on dry ice according to the biospecimen plan.

### 2.10. Ethics and Informed Consent

Owners provided written informed consent prior to any study procedures, including permission to use anonymized data and images for publication. The study protocol GERI-VITAL-DOG (version v1.0; 9 November 2024) was reviewed and approved by the Institutional Animal Care and Use Committees of both participating veterinary clinics: VEGA Veterinary Clinic, Novosibirsk (protocol VEGA-VCA-2025-014, approved 28 January 2025) and BALTO Veterinary Clinic, Novosibirsk (protocol BALTO-VCB-2025-021, approved 29 January 2025). All procedures were conducted in accordance with ARRIVE 2.0 guidelines and national regulations for companion animal research.

### 2.11. Role of the Funding Source

Activator MAF, LLC (Novosibirsk, Russia) supplied Immutalon^TM^ and provided unrestricted financial support. The sponsor did not participate in study design, conduct, data collection, statistical analysis, interpretation, or manuscript preparation. Analytical independence was maintained by the investigator institutions.

## 3. Results

### 3.1. Participants and Follow-Up

Sixty geriatric dogs were randomized (30 EF-M2; 30 placebo) and comprised the intention-to-treat (ITT) population for all efficacy analyses. All participants contributed valid accelerometry for the baseline week and week 1 windows (≥5 valid days/week; wear-time ≥ 20 h/day), and all had day 28 assessments for the co-primary endpoints. Analyses followed the prespecified windows, accelerometry quality criteria, and gatekeeping hierarchy (P1 tested before P2) defined in the protocol (Figure 1).

### 3.2. Baseline Characteristics

Groups were well balanced at enrollment (Table 1). Mean age was 12.4 ± 1.8 y, baseline objective activity averaged 144.8 ± 38.3 min/day, and distributions of sex, neuter status, and symptom flags (pain, pruritus, otitis) were comparable between groups (Table 1).

### 3.3. Co-Primary Endpoints

Objective activity at week 1 (P1): EF-M2 produced a greater increase in mean daily active minutes from baseline to week 1 than placebo (adjusted mean difference, 23.05 min/day; 95% CI, 18.16–27.94; *p* < 0.001). Group means for change were 30.0 ± 9.8 min/day with EF-M2 vs. 6.8 ± 9.4 min/day with placebo (Table 2).

Vitality composite at day 28 (P2): After P1 met the gatekeeping criterion, the prespecified test of the day 28 vitality composite showed a larger improvement with EF-M2 than placebo (adjusted mean difference, 2.01 z-score units; 95% CI, 1.52–2.50; *p* < 0.001). Mean changes were 2.71 ± 1.15 with EF-M2 vs. 0.70 ± 0.71 with placebo (Table 2).

### 3.4. Key Secondary Endpoints

At week 4, EF-M2 further increased objective activity versus baseline, exceeding placebo by 33.00 min/day (95% CI, 26.83–39.18; *p* < 0.001). Hearing sensitivity improved as reflected by a larger reduction in BAER threshold at day 28 (adjusted mean difference, −5.28 dB; 95% CI, −7.53 to −3.04; *p* < 0.001). Skin barrier metrics also favored EF-M2, with lower TEWL at day 28 (adjusted mean difference, −1.35 g/m^2^·h; 95% CI, −2.45 to −0.25; *p* = 0.02). Among symptomatic subsets, pruritus improved more with EF-M2 (PVAS adjusted mean difference, −2.05; 95% CI, −3.07 to −1.04; *p* = 0.001), whereas the otitis index (OTIS-3) did not differ between groups (adjusted mean difference, −0.64; 95% CI, −3.22 to 1.95; *p* = 0.64) (Table 3).

### 3.5. Global Impression of Change

Owner-anchored global improvement was more frequent with EF-M2. At day 28, 93.3% of EF-M2 recipients vs. 10.0% of placebo recipients met the PGIC responder definition (risk difference, 0.83; 95% CI, 0.69–0.97; *p* < 0.001). At day 56 (off-drug), responder rates were 50.0% vs. 16.7%, respectively (risk difference, 0.33; 95% CI, 0.11–0.56; *p* = 0.01) (Table 4).

### 3.6. Pharmacodynamic Biomarkers

Prespecified M2-skewing biomarkers changed in the expected directions by day 7. Versus placebo, EF-M2 increased the ARG1:iNOS ratio (adjusted mean difference, 0.196; 95% CI, 0.162–0.230; *p* < 0.001) and IL-10 (+2.85 pg/mL; 95% CI, 2.13–3.57; *p* < 0.001), while decreasing TNF-α (−1.12 pg/mL; 95% CI, −1.44 to −0.81; *p* < 0.001), hs-CRP (−0.71 mg/L; 95% CI, −0.95 to −0.46; *p* < 0.001), and IL-6 (−0.44 pg/mL; 95% CI, −0.56 to −0.32; *p* < 0.001). These early PD shifts were concordant with the clinical improvements observed at week 1 and day 28 (Table 5).

### 3.7. Safety

Through day 28, adverse events were infrequent and mostly grade 1 by VCOG-CTCAE. The proportion of dogs with ≥1 AE was 16.7% with EF-M2 and 20.0% with placebo; there were no serious AEs in either group. Injection-site reactions and transient lethargy were the most common events; low-grade transaminase elevations occurred in one dog per group (Table 6).

### 3.8. Data Completeness and Protocol Adherence

Accelerometry completeness was high across all windows (valid-day yield ≥96% per window; Table S1). The prespecified day 14 step-up rule was implemented under blinded conditions and treated as part of the randomized strategy (treatment-policy principle), consistent with the protocol, and did not affect ITT inclusion.

## 4. Discussion

Principal findings: In this randomized, double-blind, placebo-controlled trial of older companion dogs, subcutaneous Immutalon (EF-M2) improved real-world activity (co-primary endpoint at day 7) and a prespecified composite “vitality” score at day 28, with convergent, directionally favorable changes across key secondary domains (owner-reported function and appetite; objective accelerometry in week 4; and prespecified sensory and skin-barrier readouts in relevant subgroups). Safety was acceptable and indistinguishable from placebo, with no withdrawals for drug intolerance. Collectively, these results support clinically meaningful benefits of macrophage-directed immunomodulation on multidomain “inflammaging”-linked impairment in geriatric dogs. The prespecified mid-course “step-up” algorithm (day 14) was associated with numerically larger on-treatment gains in dogs meeting criteria for insufficient early response, consistent with an exposure–response relationship anticipated a priori.

Context within the literature: Our findings extend prior blinded evidence that Immutalon (EF-M2)—an analytically defined, CLEC10A-engaging, glyco-edited derivative of vitamin D-binding protein—produces dose-frequency-dependent clinical benefit and biomarker shifts in naturally occurring disease [15,21,22]. In a three-arm osteoarthritis trial (IMPAWS-OA-1), EF-M2 reduced owner-reported pain (least-squares mean ΔCBPI-PSS −2.11 with thrice-weekly dosing vs. −0.54 placebo) and improved force-plate kinetics (+7.08% body-weight peak vertical force), with parallel increases in IL-10 and in the ARG1/iNOS ratio and reductions in TNF-α; adverse events were infrequent, mild, and balanced with placebo [15]. Those data provide a mechanistic and clinical benchmark for macrophage repolarization as a disease-modifying strategy and are directionally concordant with the present multidomain “vitality” outcomes [15].

Importantly, EF-M2 represents the M2-skewing member of a “dial-a-bias” GcMAF2.0 platform in which single-monosaccharide precision (exposing α-GalNAc at Thr420) drives selective engagement of CLEC10A and reproducible IL-10/ARG1-dominant programs under low-endotoxin, GMP-traceable manufacturing—features that increase biological plausibility and limit artifactual pro-inflammatory readouts [9,14,21,22,23].

Potential mechanisms: The vitality construct used here—anchored in objective activity, owner-reported energy and appetite, and complemented by subdomain modules (e.g., BAER, TEWL)—captures clinical manifestations plausibly downstream of innate-immune tone in aged animals (“inflammaging”) [17,18,20,24,25,26]. For clinicians and owners, this means that the approximately 2-unit between-group difference in the vitality composite z-score at day 28 does not represent a marginal numerical shift. Rather, it reflects a change in which dogs receiving EF-M2, on average, moved from a typical “older dog” baseline profile into a level of energy and appetite that is clearly noticeable in day-to-day life—for example, more spontaneous walking and play, greater engagement with the household, and more consistent completion of meals. EF-M2 is designed to bias monocyte–macrophage state toward an IL-10/ARG1-rich phenotype via α-GalNAc ligation of the macrophage lectin CLEC10A (CD301) [9,21], a Ca^2+^-dependent receptor whose clustering initiates SYK-centered signaling [27,28] and, in IL-4/IL-13–conditioned milieus, consolidates STAT6–PPARγ programs that favor efferocytosis, matrix repair, and nociceptor desensitization [29,30,31,32]. The tight correspondence between clinical benefit and macrophage polarity reported in osteoarthritis—ARG1/iNOS increases tracking pain relief and gait restoration—offers a biologically coherent template for interpreting the present multidomain improvements and motivated the same serum PD panel (ARG1/iNOS, IL-10, TNF-α) in our protocol [15].

Manufacturing advances that define EF-M2 at the single-glycan level (intact-protein LC-MS fingerprint, endotoxin ≤ 0.05 EU mg^−1^, CLEC10A-binding potency) reduce mechanistic ambiguity and support the inference that the observed clinical effects reflect receptor-proximal biology rather than contaminants or lot heterogeneity [22,23].

In our mechanistic framework, these serum biomarker shifts are expected to contribute to alleviating “sickness-behavior” features of inflammaging—such as fatigue, reduced spontaneous activity, and diminished appetite—by reducing tonic TNF-α/IL-6 signaling and increasing IL-10/ARG1-associated resolution programs [1,2,3,4,5,6,7]. The temporal pattern in this trial, with early changes in IL-10, ARG1/iNOS, and pro-inflammatory markers by day 7 paralleling improvements in daily activity and vitality scores, is consistent with this model. However, the study was not powered to formally test mediation, and we cannot infer that the biomarker changes are exclusively or directly responsible for the observed clinical benefits; rather, they provide supportive evidence of a coherent immunological mechanism.

Rationale for the “step-up” strategy: The protocol implemented a blinded, prespecified “step-up” at day 14 (q72 h → q48 h) for dogs with <25% early improvement in their profile-defining domain. This was intended to preserve receptor engagement in animals with slower pharmacodynamic onset and is aligned with prior evidence that thrice-weekly EF-M2 outperforms twice-weekly dosing across pain, function, and macrophage biomarkers—an exposure–response relationship consistent with CLEC10A’s rapid internalization/recycling kinetics and the need to maintain M2 dominance against homeostatic counter-signals [15,22,33].

The observed clinical gains after step-up are therefore mechanistically plausible within a model that links dosing cadence to sustained macrophage repolarization [15,33].

Clinical and translational implications: For geriatric dogs, rapid improvement in free-living activity at one week and a broader vitality signal at four weeks suggest that EF-M2 may offer a practical, non-sedating option to ameliorate low-grade, inflammation-linked functional decline, with favorable tolerability and without the gastrointestinal, renal, or hepatic constraints that limit long-term NSAID use [34,35,36]. Although our study was not designed around a single organ system, prespecified sensory and barrier modules (BAER, TEWL) allow hypothesis generation around neurosensory and dermatologic dimensions of inflammaging and may help target phenotypes for which macrophage repolarization is most impactful [20,26]. Because companion dogs share environmental exposures with humans and permit integration of owner-reported outcomes with objective activity monitors and serum PD markers, the present data, together with blinded, placebo-controlled osteoarthritis findings, support the usefulness of this species as a translational bridge for macrophage-modulating interventions [37,38,39].

Strengths and limitations: Strengths include (1) a randomized, double-blind, placebo-controlled design with mirror-image injections to protect masking; (2) prospectively defined co-primary endpoints and a hierarchical analysis plan; (3) objective activity monitoring with prespecified wear-time and validity criteria; and (4) incorporation of mechanism-anchored serum biomarkers grounded in an external evidence base.

Several limitations deserve emphasis. First, the sample size, while powered for the co-primary endpoints, limits precision for secondary/safety outcomes and for subgroup analyses (e.g., sensory or skin-barrier modules). Second, the 8-week horizon (4-week dosing plus 4-week off-drug) is too short to assess durability beyond the immediate pharmacodynamic window or to evaluate structural or cognitive trajectories; longer studies will be required. Third, the protocolized day 14 step-up algorithm introduces heterogeneity in dose frequency within the EF-M2 group. Because this escalation was prespecified, applied under full blinding, and mirrored in the placebo arm, it does not compromise randomization, but it does mean that our estimates reflect the effectiveness of a treatment strategy (“q72 h with step-up in partial responders”) rather than a single fixed-frequency regimen, consistent with the treatment-policy principle used in the ITT analyses. Fourth, serum PD markers provide systemic, not tissue-localized, readouts; consequently, although the observed pattern is consistent with a shift toward an IL-10/ARG1-dominated macrophage profile, we cannot directly infer the immune state within specific target tissues such as joints, skin, or the nervous system. Future work incorporating synovial, dermal, or neural fluid/tissue sampling (where feasible) will be required to map the concordance between systemic and local immune changes.

Additional limitations relate to owner-reported outcomes and external validity. As in all owner-reported domains, expectancy or placebo effects cannot be fully excluded, even though owners were fully blinded to treatment status, had no access to allocation codes, and observed indistinguishable injection procedures in both arms. The alignment of these outcomes with objective accelerometry and sensory modules (BAER, TEWL) mitigates, but does not eliminate, this concern. In terms of external validity, both recruiting sites were referral veterinary clinics located in a single metropolitan area (Novosibirsk, Russia). As a result, enrolled dogs shared a relatively homogeneous set of environmental exposures, husbandry practices, and breed distributions, which may not fully reflect geriatric dog populations in other geographic regions or practice settings. The generalizability of these findings, therefore, warrants confirmation in larger, more diverse multicenter cohorts that include dogs from different climates, lifestyles, and healthcare systems.

Future directions: Three paths follow directly from these data and the external mechanistic literature. (1) Duration and durability. A 3–6-month, multicenter trial with sustained dosing (e.g., 3×-weekly induction followed by 2×-weekly maintenance) should quantify durability, define optimal cadence, and embed sparse PK/PD sampling to relate exposure to ARG1/iNOS inflection. (2) Phenotype targeting. Enrich future cohorts for prespecified symptom constellations (e.g., low energy + reduced activity ± dermatologic or sensory comorbidity) and prospectively validate PD–clinical coupling as a stratification tool. (3) Combination and comparators. Evaluate EF-M2 within multimodal geriatric-care programs (nutrition, activity, rehabilitation), and in disease-specific contexts (e.g., painful osteoarthritis or pruritic dermatoses), consider head-to-head or add-on designs against standard agents to quantify incremental value and safety. Mechanistic substudy arms—including CLEC10A occupancy and macrophage-transcriptome panels—would further anchor causality to receptor-proximal events.

## 5. Conclusions

In this multicenter, randomized, double-blind, placebo-controlled study of client-owned geriatric dogs, subcutaneous EF-M2 (Immutalon) achieved the two prespecified co-primary endpoints—an early increase in device-measured daily activity at day 7 and a sustained improvement in a validated vitality composite at day 28—over matched placebo. These gains were accompanied by favorable changes across key secondary domains and were maintained, in attenuated form, during the off-drug observation through day 56, while the overall safety profile was indistinguishable from that of the control. Together, these data demonstrate that brief, outpatient administration of EF-M2 can produce rapid, durable, and clinically meaningful improvements in real-world function and owner-perceived well-being in aged dogs, consistent with the a priori statistical analysis plan and endpoint hierarchy specified in the protocol.

The clinical benefits were paralleled by pharmacodynamic evidence of macrophage repolarization toward an M2-dominant state (increase in the ARG1/iNOS ratio with reciprocal IL-10↑/TNF-α↓), supporting a coherent mechanism in which precision glyco-modulation of the CLEC10A axis re-wires myeloid tone to relieve sickness behavior and improve multi-domain function. This mechanistic link is aligned with contemporary, analytically defined GcMAF2.0 biology—of which EF-M2 is an M2-biased variant—spanning receptor biophysics, primary-cell transcriptomics, and cross-species in vivo signals.

The direction and durability of the present effects also accord with randomized, placebo-controlled evidence in spontaneous canine osteoarthritis, where EF-M2 yielded dose-frequency–dependent reductions in pain, objective gains in locomotor function, and concordant shifts in macrophage-related serum biomarkers without excess adverse events.

While longer follow-up and imaging-anchored studies are required to define structural modification and rare toxicities, the current findings provide rigorous proof-of-concept that targeted macrophage modulation with EF-M2 can rapidly improve objective activity and owner-relevant vitality in the aging companion animal setting. These data establish a translational platform for exposure–response optimization and for testing EF-M2—alone or in multimodal regimens—in chronic, low-grade inflammatory states where macrophage immunometabolism is believed to modulate clinical outcomes.

## Figures and Tables

**Table 1:** Baseline characteristics at enrollment (ITT population).

Variable	Placebo (n = 30)	EF-M2 (n = 30)	Total (n = 60)	*p* Value
Age, y	12.3 (1.7)	12.6 (1.9)	12.4 (1.8)	0.48
Weight, kg	17.3 (10.3)	18.6 (11.7)	17.9 (11.0)	0.65
Active minutes/day at baseline	147.5 (43.9)	142.2 (32.2)	144.8 (38.3)	0.60
CBPI-PSS (0–10)	3.84 (2.52)	4.68 (2.03)	4.26 (2.31)	0.16
HRQL-vitality (0–10)	3.79 (1.40)	3.98 (1.06)	3.88 (1.23)	0.56
Appetite VAS (0–10)	6.62 (1.54)	6.50 (1.41)	6.56 (1.47)	0.74
Male sex—n (%)	17 (56.7%)	15 (50.0%)	32 (53.3%)	0.80
Intact—n (%)	12 (40.0%)	15 (50.0%)	27 (45.0%)	0.60
Pain at baseline—n (%)	20 (66.7%)	16 (53.3%)	36 (60.0%)	0.19
Pruritus at baseline—n (%)	11 (36.7%)	8 (26.7%)	19 (31.7%)	0.58
Otitis at baseline—n (%)	4 (13.3%)	7 (23.3%)	11 (18.3%)	0.50

Data are mean (SD) or n (%). *p*-values from Welch’s *t*-test (continuous) or χ^2^ test (categorical). Abbreviations: CBPI-PSS—Canine Brief Pain Inventory–Pain Severity Score; HRQL—Health-Related Quality of Life; VAS—Visual Analog Scale.

**Table 2:** Co-primary endpoints.

Endpoint (Change from Baseline)	Placebo (n = 30)	EF-M2 (n = 30)	Adjusted Difference (EF-M2 − Placebo), 95% CI	*p* Value
P1: Objective activity (week 1 vs. Baseline), min/day—Δ mean (SD)	6.8 (9.4)	30.0 (9.8)	23.05 (18.16 to 27.94)	<0.001
P2: Vitality composite z (day 28 vs. Day 0)—Δ mean (SD)	0.70 (0.71)	2.71 (1.15)	2.01 (1.52 to 2.50)	<0.001

Higher values reflect improvement in activity and vitality. Vitality composite z is the sum of domain-wise z-scores standardized to the day 0 distribution (CBPI-PSS inverted), per protocol.

**Table 3:** Key secondary endpoints.

Endpoint (Change from Baseline)	Placebo	EF-M2	Adjusted Difference (EF-M2 − Placebo), 95% CI	*p* Value
Objective activity (week 4 vs. Baseline), min/day—Δ mean (SD)	9.9 (12.3)	43.0 (11.9)	33.00 (26.83 to 39.18)	<0.001
BAER threshold (dB), day 28 − day 0—Δ mean (SD)	−0.92 (5.53)	−4.78 (5.22)	−5.28 (−7.53 to −3.04)	<0.001
TEWL (g/m^2^·h), day 28 − day 0—Δ mean (SD)	−1.83 (2.75)	−3.57 (3.52)	−1.35 (−2.45 to −0.25)	0.02
PVAS (0–10), day 28 − day 0—Δ mean (SD) [symptomatic at baseline] *	−0.85 (1.04)	−2.96 (1.28)	−2.05 (−3.07 to −1.04)	0.001
OTIS-3 (0–10), day 28 − day 0—Δ mean (SD) [symptomatic at baseline] †	−3.00 (2.16)	−3.71 (1.89)	−0.64 (−3.22 to 1.95)	0.64

* PVAS subset: Placebo n = 11, EF-M2 n = 8. † OTIS-3 subset: Placebo n = 4, EF-M2 n = 7. Endpoint definitions and analysis windows per protocol.

**Table 4:** Patient/owner-anchored global impression of change (PGIC responders).

Outcome	Placebo	EF-M2	Risk Difference (95% CI)	*p* Value
PGIC responder (day 28)—n/N (%)	3/30 (10.0%)	28/30 (93.3%)	0.83 (0.69 to 0.97)	<0.001
PGIC responder (day 56, off-drug)—n/N (%)	5/30 (16.7%)	15/30 (50.0%)	0.33 (0.11 to 0.56)	0.01

*p*-values from Fisher’s exact test. Anchoring definition is prespecified in the protocol.

**Table 5:** Pharmacodynamic biomarkers (Δ day 7 − day 0).

Biomarker—Δ Mean (SD)	Placebo	EF-M2	Adjusted Difference (EF-M2 − Placebo), 95% CI	*p* Value
ARG1:iNOS ratio	0.070 (0.055)	0.264 (0.078)	0.196 (0.162 to 0.230)	<0.001
IL-10, pg/mL	1.40 (0.97)	4.26 (1.73)	2.85 (2.13 to 3.57)	<0.001
TNF-α, pg/mL	−0.27 (0.48)	−1.40 (0.72)	−1.12 (−1.44 to −0.81)	<0.001
hs-CRP, mg/L	−0.15 (0.36)	−0.85 (0.57)	−0.71 (−0.95 to −0.46)	<0.001
IL-6, pg/mL	−0.07 (0.22)	−0.51 (0.25)	−0.44 (−0.56 to −0.32)	<0.001

Positive differences reflect greater increases; negative differences reflect greater decreases vs. placebo. All models adjust for baseline (day 0).

**Table 6:** Safety overview (safety population = ITT; on-treatment through day 28).

Safety Outcome	Placebo (n = 30)	EF-M2 (n = 30)
Subjects with ≥1 adverse event—n (%)	6 (20.0%)	5 (16.7%)
Total adverse events—n	6	5
Grade ≥ 2 AEs—n	1	1
Serious AEs—n	0	0
Injection site erythema—n (%)	3 (10.0%)	1 (3.3%)
Mild lethargy ≤ 24 h—n (%)	2 (6.7%)	2 (6.7%)
Transient low-grade fever—n (%)	0 (0.0%)	1 (3.3%)
ALT/AST increased—n (%)	1 (3.3%)	1 (3.3%)

Event grading by VCOG-CTCAE. Laboratory ALT/AST were also summarized at day 28; no clinically meaningful between-group differences were observed.

## Data Availability

The raw data supporting the conclusions of this article will be made available by the authors on request.

## Supplementary Materials

The following supporting information can be downloaded at: https://www.mdpi.com/article/10.3390/vetsci12121168/s1, Table S1: The minimal dataset for *GERI-VITAL-DOG*.

## References

[1] Fulop T., Larbi A., Pawelec G., Khalil A., Cohen A.A., Hirokawa K., Witkowski J.M., Franceschi C. (2023). Immunology of Aging: The Birth of Inflammaging. Clin. Rev. Allergy Immunol..

[2] De Maeyer R.P.H., Chambers E.S. (2021). The impact of ageing on monocytes and macrophages. Immunol. Lett..

[3] Linehan E., Fitzgerald D.C. (2015). Ageing and the immune system: Focus on macrophages. Eur. J. Microbiol. Immunol..

[4] McCusker R.H., Kelley K.W. (2013). Immune-neural connections: How the immune system’s response to infectious agents influences behavior. J. Exp. Biol..

[5] Chen O., Luo X., Ji R.R. (2023). Macrophages and microglia in inflammation and neuroinflammation underlying different pain states. Med. Rev..

[6] Chen S., Saeed A.F.U.H., Liu Q., Jiang Q., Xu H., Xiao G.G., Rao L., Duo Y. (2023). Macrophages in immunoregulation and therapeutics. Signal Transduct. Target. Ther..

[7] Nguyen T.Q.T., Cho K.A. (2025). Targeting immunosenescence and inflammaging: Advancing longevity research. Exp. Mol. Med..

[8] Zaal A., Li R.J.E., Lübbers J., Bruijns S.C.M., Kalay H., van Kooyk Y., van Vliet S.J. (2020). Activation of the C-Type Lectin MGL by Terminal GalNAc Ligands Reduces the Glycolytic Activity of Human Dendritic Cells. Front. Immunol..

[9] van Vliet S.J., van Liempt E., Saeland E., Aarnoudse C.A., Appelmelk B., Irimura T., Geijtenbeek T.B., Blixt O., Alvarez R., van Die I. (2005). Carbohydrate profiling reveals a distinctive role for the C-type lectin MGL in the recognition of helminth parasites and tumor antigens by dendritic cells. Int. Immunol..

[10] Marcelo F., Supekar N., Corzana F., van der Horst J.C., Vuist I.M., Live D., Boons G.P.H., Smith D.F., van Vliet S.J. (2019). Identification of a secondary binding site in human macrophage galactose-type lectin by microarray studies: Implications for the molecular recognition of its ligands. J. Biol. Chem..

[11] Gabba A., Bogucka A., Luz J.G., Diniz A., Coelho H., Corzana F., Cañada F.J., Marcelo F., Murphy P.V., Birrane G. (2021). Crystal Structure of the Carbohydrate Recognition Domain of the Human Macrophage Galactose C-Type Lectin Bound to GalNAc and the Tumor-Associated Tn Antigen. Biochemistry.

[12] Rehder D.S., Nelson R.W., Borges C.R. (2009). Glycosylation status of vitamin D binding protein in cancer patients. Protein Sci..

[13] Nabeshima Y., Abe C., Kawauchi T., Hiroi T., Uto Y., Nabeshima Y.I. (2020). Simple method for large-scale production of macrophage activating factor GcMAF. Sci. Rep..

[14] Borges C.R., Rehder D.S. (2016). Glycan structure of Gc Protein-derived Macrophage Activating Factor as revealed by mass spectrometry. Arch. Biochem. Biophys..

[15] Pokushalov E., Kudlay D., Revkov N., Shcherbakova A., Johnson M., Miller R. (2025). Targeted Macrophage Modulation as a Disease-Modifying Approach in Canine Osteoarthritis: The Efficacy of EF-M2 (Immutalon) in a Double-Blind Placebo-Controlled Study. Vet. Sci..

[16] Strizova Z., Benesova I., Bartolini R., Novysedlak R., Cecrdlova E., Foley L.K., Striz I. (2023). M1/M2 macrophages and their overlaps-myth or reality?. Clin. Sci..

[17] Brown D.C., Boston R.C., Coyne J.C., Farrar J.T. (2008). Ability of the canine brief pain inventory to detect response to treatment in dogs with osteoarthritis. J. Am. Vet. Med. Assoc..

[18] Davies V., Reid J., Wiseman-Orr M.L., Scott E.M. (2019). Optimising outputs from a validated online instrument to measure health-related quality of life (HRQL) in dogs. PLoS ONE.

[19] Wiseman-Orr M.L., Scott E.M., Reid J., Nolan A.M. (2006). Validation of a structured questionnaire as an instrument to measure chronic pain in dogs on the basis of effects on health-related quality of life. Am. J. Vet. Res..

[20] Yam P.S., Penpraze V., Young D., Todd M.S., Cloney A.D., Houston-Callaghan K.A., Reilly J.J. (2011). Validity, practical utility and reliability of Actigraph accelerometry for the measurement of habitual physical activity in dogs. J. Small Anim. Pract..

[21] Szczykutowicz J. (2023). Ligand Recognition by the Macrophage Galactose-Type C-Type Lectin: Self or Non-Self?—A Way to Trick the Host’s Immune System. Int. J. Mol. Sci..

[22] Ravnsborg T., Olsen D.T., Thysen A.H., Christiansen M., Houen G., Højrup P. (2010). The glycosylation and characterization of the candidate Gc macrophage activating factor. Biochim. Biophys. Acta.

[23] Kirikovich S.S., Levites E.V., Proskurina A.S., Ritter G.S., Peltek S.E., Vasilieva A.R., Ruzanova V.S., Dolgova E.V., Oshihmina S.G., Sysoev A.V. (2023). The Molecular Aspects of Functional Activity of Macrophage-Activating Factor GcMAF. Int. J. Mol. Sci..

[24] Brown D.C., Boston R.C., Coyne J.C., Farrar J.T. (2007). Development and psychometric testing of an instrument designed to measure chronic pain in dogs with osteoarthritis. Am. J. Vet. Res..

[25] Hilborn E.C., Rudinsky A.J., Kieves N.R. (2024). Commercially available wearable health monitors in dogs only had a very strong correlation during longer durations of time: A pilot study. Am. J. Vet. Res..

[26] Stanger A., Buhmann G., Dörfelt S., Zablotski Y., Fischer A. (2024). Rapid hearing threshold assessment with modified auditory brainstem response protocols in dogs. Front. Vet. Sci..

[27] Kurashina R., Denda-Nagai K., Saba K., Hisai T., Hara H., Irimura T. (2021). Intestinal lamina propria macrophages upregulate interleukin-10 mRNA in response to signals from commensal bacteria recognized by MGL1/CD301a. Glycobiology.

[28] Sancho D., Reis e Sousa C. (2012). Signaling by myeloid C-type lectin receptors in immunity and homeostasis. Annu. Rev. Immunol..

[29] Szanto A., Balint B.L., Nagy Z.S., Barta E., Dezso B., Pap A., Szeles L., Poliska S., Oros M., Evans R.M. (2010). STAT6 transcription factor is a facilitator of the nuclear receptor PPARγ-regulated gene expression in macrophages and dendritic cells. Immunity.

[30] Yoon Y.S., Kim S.Y., Kim M.J., Lim J.H., Cho M.S., Kang J.L. (2015). PPARγ activation following apoptotic cell instillation promotes resolution of lung inflammation and fibrosis via regulation of efferocytosis and proresolving cytokines. Mucosal Immunol..

[31] Yin C., Heit B. (2021). Cellular Responses to the Efferocytosis of Apoptotic Cells. Front. Immunol..

[32] Celik M.Ö., Labuz D., Keye J., Glauben R., Machelska H. (2020). IL-4 induces M2 macrophages to produce sustained analgesia via opioids. JCI Insight.

[33] Heger L., Balk S., Lühr J.J., Heidkamp G.F., Lehmann C.H.K., Hatscher L., Purbojo A., Hartmann A., Garcia-Martin F., Nishimura S.I. (2018). CLEC10A Is a Specific Marker for Human CD1c+ Dendritic Cells and Enhances Their Toll-Like Receptor 7/8-Induced Cytokine Secretion. Front. Immunol..

[34] Pye C., Bruniges N., Peffers M., Comerford E. (2022). Advances in the pharmaceutical treatment options for canine osteoarthritis. J. Small Anim. Pract..

[35] FDA Center for Veterinary Medicine (2022). Get the Facts About Pain Relievers for Pets.

[36] Homedes J., Ocak M., Riedle S., Salichs M. (2024). A blinded, randomized and controlled multicenter field study investigating the safety and efficacy of long-term use of enflicoxib in the treatment of naturally occurring osteoarthritis in client-owned dogs. Front. Vet. Sci..

[37] Kaeberlein M., Creevy K.E., Promislow D.E. (2016). The dog aging project: Translational geroscience in companion animals. Mamm. Genome.

[38] Hoffman J.M., Creevy K.E., Franks A., O’Neill D.G., Promislow D.E.L. (2018). The companion dog as a model for human aging and mortality. Aging Cell.

[39] Creevy K.E., Akey J.M., Kaeberlein M., Promislow D.E.L. (2022). Dog Aging Project Consortium. An open science study of ageing in companion dogs. Nature.

